# Rapamycin reduces DNA damage of in vitro matured oocytes by promoting *Rad51*-mediated homologous recombination

**DOI:** 10.1186/s12958-025-01428-6

**Published:** 2025-07-03

**Authors:** Qiyu Yang, Ying Chen, Jiayu Huang, Junying Tang, Lixia Zhu, Jingyu Li, Chao Tong

**Affiliations:** 1 Department of Obstetrics and Gynecology, The First Affiliated Hospital of Chongqing Medical University, No.1 Youyi Road, Yuzhong District, Chongqing, 400016 China; 2 Chongqing Medical University, No.1 Yixueyuan Road, Yuzhong District, Chongqing, 400016 China; 3 Reproductive Medicine Center, Tongji Hospital, Tongji Medical College, Huazhong University of Science and Technology, No.1095, Jiefang Road, Wuhan, 430030 China; 4 Chongqing Key Laboratory of Human Embryo Engineering and Precision Medicine, Center for Reproductive Medicine, Women and Children’s Hospital of Chongqing Medical University, No. 64, Jintang Road, Chongqing, 400013 China; 5 National Clinical Research Center for Child Health and Disorders, Ministry of Education Key Laboratory of Child Development and Disorders, Children’s Hospital of Chongqing Medical University, No. 20, Jinyu Road, Yubei District, Chongqing, 401120 China

**Keywords:** Rapamycin, In vitro maturation, Oocyte, DNA damage, Homologous recombination

## Abstract

**Background:**

The quality of in vitro matured (IVM) oocytes remains inferior to that of in vivo matured oocytes, likely due to DNA damage induced by the in vitro environment. Although rapamycin has been shown to improve the developmental competence of IVM oocytes by reducing DNA double-strand breaks (DSBs), the underlying mechanisms remain unclear.

**Methods:**

Immature oocytes from 6 ~ 8-week-old ICR female mice were subjected to IVM with or without rapamycin. After 14 ~ 16 h, the maturation rate, DNA DSB levels, and subsequent developmental competence of IVM oocytes were assessed. The homologous recombination (HR) and non-homologous end joining (NHEJ) pathways were selectively inhibited using RAD51 and DNA-PK inhibitors, respectively, to elucidate the predominant DNA damage repair pathway during IVM and investigate the effects of rapamycin on this process.

**Results:**

Embryos derived from the rapamycin-treated group exhibited significantly higher 2PN and cleavage rates compared to the control group. MII oocytes cultured with rapamycin showed reduced γH2AX immunofluorescence intensity, indicating lower DNA damage levels. Additionally, the expression levels of RAD51 and DNAPK were elevated in rapamycin-treated oocytes. Inhibition of RAD51 significantly reduced the maturation rate and increased DNA damage levels, whereas DNAPK inhibition had no effect on oocyte development or quality. Importantly, the beneficial effects of rapamycin on IVM oocytes were diminished under RAD51 inhibition.

**Conclusion:**

Rapamycin improves the developmental competence of IVM oocytes by enhancing the RAD51-mediated HR pathway, thereby enhancing DNA stability.

**Supplementary Information:**

The online version contains supplementary material available at 10.1186/s12958-025-01428-6.

## Background

Oocyte in vitro maturation (IVM), which refers to culturing the immature oocytes in a specific medium until the extrusion of first polar body (PB1), is a rapidly developing technology of reproductive medicine in the past three decades [1]. Since the culture system supporting IVM of human oocytes was established in 1965 [2, 3], improving maturation rates and quality of IVM oocytes has been the focus of many researchers. To date, considerable progress has been made in the optimization of the culture system and medium components, however, the quality of IVM oocytes remains inferior to that of in vivo oocytes [4, 5].

The damages from in vitro environment might be the reason, which adversely affects oocyte maturation, primarily through metabolic and genetic disruptions [6]. Specifically, the Krebs cycle is impaired due to downregulation of key enzymes (ACAT1, HADHA) in IVM oocytes, reducing acetyl-CoA and succinate production, which weakens energy metabolism. Besides, calcium signaling disruption further diminishes enzyme activity, exacerbating metabolic inefficiency. Although compensatory upregulation of nicotinamide nucleotide transhydrogenase (NNT) partially offsets energy deficits by converting NADPH to NADH, this mechanism may inadvertently elevate oxidative stress, contributing to mitochondrial dysfunction and chromosomal instability. Based on our previous study, IVM oocytes presented higher levels of DNA double-strand breaks (DSB), compared with the in vivo matured ones. Notably, rapamycin, a macrolide metabolite produced by *Streptomyces hygroscopicus*, could markedly improve the developmental competence of IVM oocytes by reducing their DNA DSB levels, both in mice and humans [7, 8].

Rapamycin exerts its biological effects primarily by inhibiting the mammalian target of rapamycin (mTOR) pathway, a key regulator of oocyte meiosis [9] and embryonic development [10]. Intriguingly, while conditional *Mtor* knockout or high-dose rapamycin treatment impairs oocyte quality and developmental competence, lower rapamycin concentrations have been shown to exert beneficial effects [7, 9]. This dose-dependent dichotomy may stem from the multifaceted roles of the mTOR pathway in cellular processes, including its complex regulatory networks and potential negative feedback mechanisms [11, 12]. In our previous study, we identified 10 nM as the optimal rapamycin concentration for enhancing oocyte IVM outcomes through dose-response experiments [7]. However, the underlying mechanism by which 10 nM rapamycin reduces DNA DSB levels in oocytes remains to be elucidated.

Most studies regarding rapamycin and DNA damage were based on cancer cells. Several studies demonstrated that rapamycin can promote DNAPK-mediated non-homologous end joining (NHEJ) to repair DNA DSB through mTOR/AKT pathway [13–15], while other studies showed that rapamycin negatively affected the recruitment of BRCA1 and RAD51 at the DNA damage site, leading to the inhibition of both homologous recombination (HR) and NHEJ [16]. These contradictory results in cancer cells could not be applied to the oocytes, therefore, the current study aims to confirm the protective effect of rapamycin on the oocytes matured in vitro, clarify the main DSB-repair pathway during IVM, and explore the impact of rapamycin on the critical molecules in HR or NHEJ.

## Methods

### Ethics approval

All the experimental procedures for animals were conducted following the guidelines and approved by the Ethical Committee of First Affiliated Hospital of Chongqing Medical University (IACUC-CQMU-2023-0422).

### Immature mice oocyte collection and in vitro maturation

ICR female mice aged 6 ~ 8 weeks were purchased from Beijing Vital River Laboratory Animal Technology Co., Ltd. Specifically, the bilateral ovaries were isolated, transferred into M2 medium (EasyCheck, Nanjing, China), and fragmented to release immature GV oocytes. After being washed five times in M2 medium, the denuded GV oocytes were moved to the G1-plus medium (Vitrolife, Sweden), with or without 10 nM rapamycin (MedChemExpress, New Jersey, USA), and then cultured for 16 h at 37 °C under 5% CO_2_ in humidified air. Following IVM, MII oocytes were recognized with the extrusion of the PB1. IBR2 (MedChemExpress, New Jersey, USA) was used to inhibit RAD51, and LTURM34 (MedChemExpress, New Jersey, USA) was used to inhibit DNAPK.

### Intracytoplasmic sperm injection (ICSI) for IVM mice oocytes

A Piezo sperm injection instrument (PiezoXpert, Eppendorf, Germany) was used for ICSI. The injection pipettes had an internal diameter of 8–10 μm at the tip and were siliconized before use. The sperm tails were removed by Piezo pulses, and only one randomly selected sperm head was injected into each oocyte. After 15 min of recovery, the surviving zygotes were washed three times in KSOM medium (Sigma-Aldrich, USA) covered with mineral oil and incubated at 37 °C with 5% CO_2_ in the air. Six hours after injection, pronucleus formation was examined. The cleavage rate and blastocyst formation rate were evaluated at 48 h and 108 h, respectively.

### Immunofluorescence analysis of IVM mice oocytes

The immunofluorescence staining procedures were slightly modified based on the previous process [7]. The fixed IVM oocytes were permeabilized with 0.1% Triton X-100 (Solarbio, T8200) for 10 min, blocked for 1 h in 3% BSA, and incubated overnight at 4 °C with primary antibodies. The chromosome was stained using Hoechst 33,258 (1:500, Servicebio, Wuhan, China). DNA DSB levels were evaluated using phospho-histone H2AX antibody (1:200, CST, Boston, USA). Fifteen to twenty-five oocytes in each group were observed and photographed under a laser scanning confocal microscope (SP8, Leica, Germany) using fixed microscopic parameters. Image J software (NIH, Bethesda, MD, USA) was applied to measure the fluorescence intensities.

### Real-time quantitative PCR

The PCR was performed as previously described [7]. For each sample, 15–20 oocytes were used to extract total mRNA with NucleoZol reagent (Macherey-Nagel, Germany). Reverse transcription of the extracted mRNA (averagely 3370 ng) was performed using HiScript II Q RT SuperMix for qPCR (+ gDNA wiper) (Vazyme, R223-01). The reaction mixture contained 10 µl ChamQ Universal SYBR qPCR Master Mix (Vazyme, Q711-02/03), 1 µl PCR Forward Primer (10 µM), 1 µl PCR Reverse Primer (10 µM), 1 µl prepared cDNA (41.7 ng/µl), and 7 µl RNase-free water. The primer pairs for real-time PCR are listed in Supplemental Table 1. PCR specificity was identified by analyzing melting curve data and gene expressions were normalized by comparison to the GAPDH transcriptional level.

### Statistical analyses

Data from at least three replicates were expressed as mean ± SEM and analyzed by T-test using GraphPad Prism 8.0 (San Diego, CA, USA). *P* < 0.05 was considered to be statistically significant.

## Results

### The developmental competence of IVM oocytes following ICSI was improved by rapamycin in mice

**Fig. 1:**
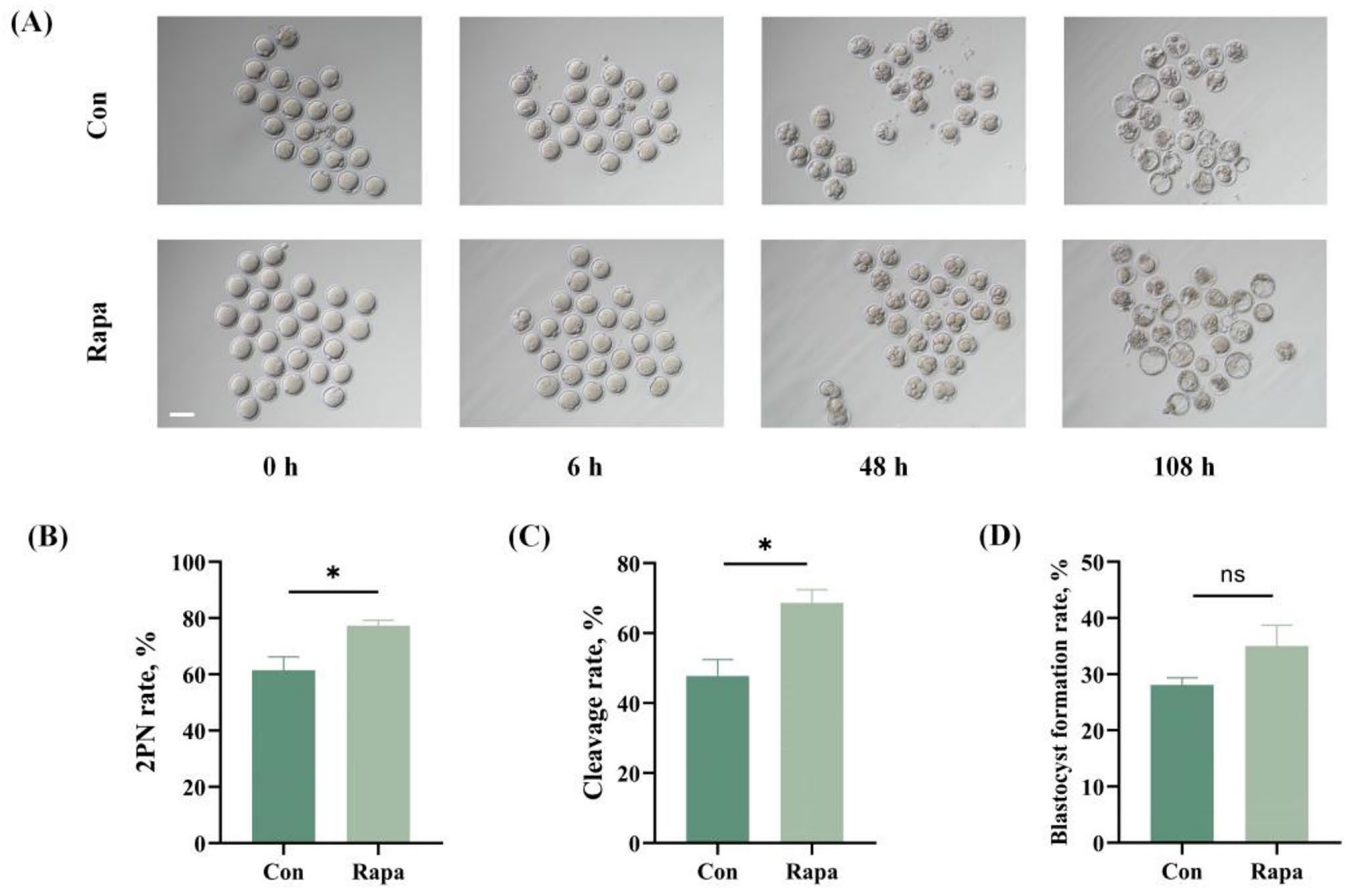
The development after ICSI of IVM mice oocytes from the control and rapamycin group. (**A**) The images of IVM oocytes at different time points after ICSI. Scale bar: 50 μm. (**B-D**) Comparison of the 2PN rates, cleavage rates, and blastocyst formation rates between the control and rapamycin groups. *P < 0.05

Based on the previous dose-effect experiment [7], 10 nM was selected as the optimal rapamycin concentration for improving IVM results. In the current study, we further observed the effect of rapamycin on the developmental competence of IVM oocytes following ICSI. The results showed that the 2PN rate and cleavage rate of oocytes from the rapamycin group were significantly increased, compared with those from the control group (2PN rate: 77.3%±3.2% vs. 61.4%±8.3%, *P* = 0.0363; cleavage rate: 68.6%±6.6% vs. 47.8%±8.1%, *P* = 0.0255). The blastocyst formation rates in the two groups presented no significant difference (35.0%±6.4% vs. 28.1%±2.2%, *P* = 0.1537), as shown in Fig. 1.

### DNA DSB in IVM oocytes were rescued by 10 nM rapamycin without activating autophagy

**Fig. 2:**
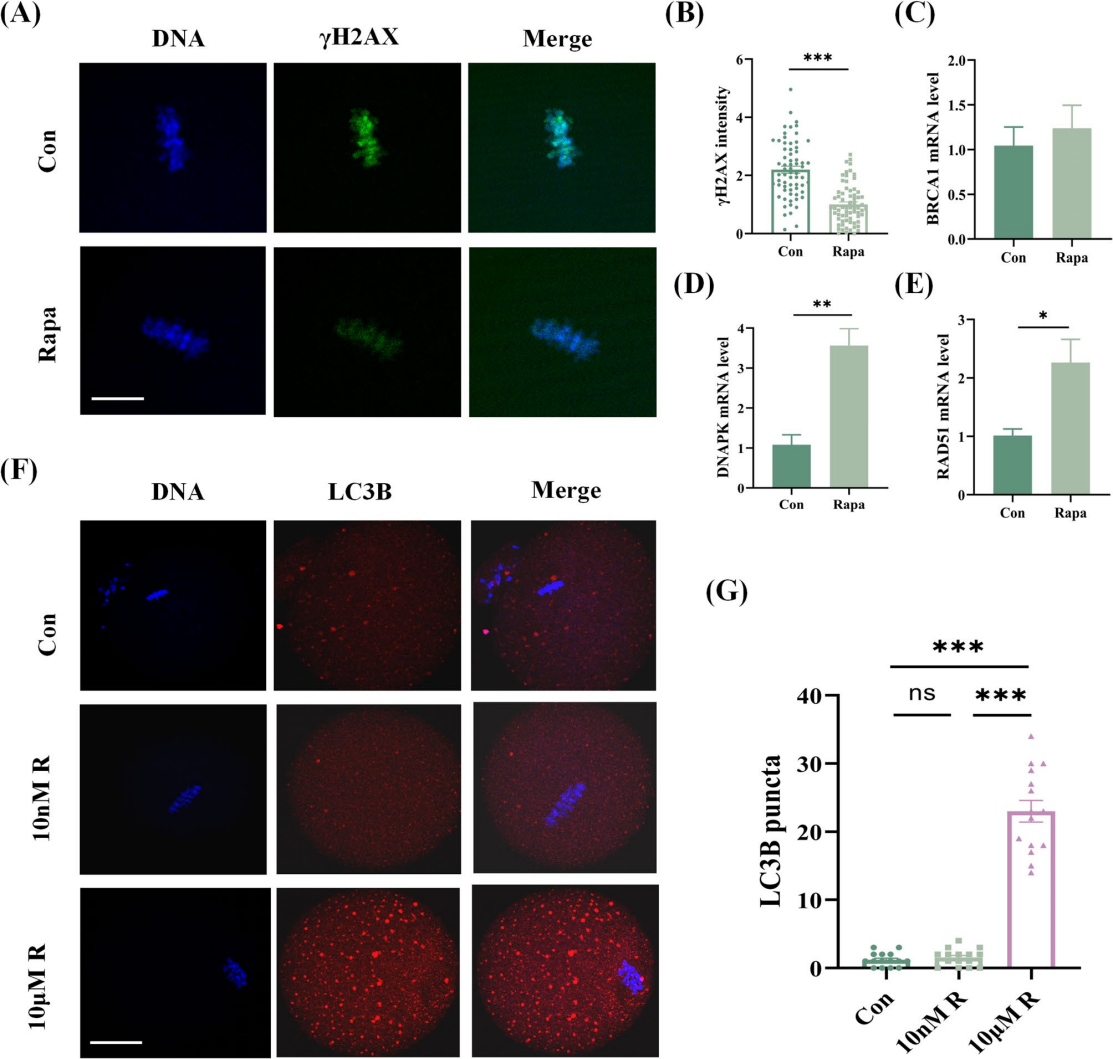
The impact of rapamycin on the DNA damage response and autophagy level in IVM oocytes. (**A**) The fluorescence images of γH2AX in IVM oocytes from the control and rapamycin groups. Scale bar: 10 μm. (**B**) The comparison of γH2AX fluorescence intensity between the two groups, ***P < 0.001. (**C-E**) The mRNA levels of the HR-associated genes *Brca1* and *Rad51* and the NHEJ-associated gene *Dnapk* in the two groups. **P < 0.01. (**F-G**) The fluorescence images of LC3B in IVM oocytes and comparison of LC3B puncta among the control, 10 nM rapamycin, and 10µM rapamycin groups. Scale bar: 25 μm, ***P < 0.001

To clarify the mechanism of rapamycin improving the developmental competence of IVM oocytes, we previously proved that rapamycin can rescue the DNA DSB in oocytes during IVM. Considering the well-known autophagy-activation effect of rapamycin, we checked the LC3B puncta in IVM oocytes. As shown in Fig. 2, the numbers of LC3B puncta in oocytes treated with 10 nM rapamycin were similar to those in the control group (1.5 ± 0.3 vs. 1.2 ± 0.3, *P* = 0.4284), while oocytes treated with 10 µM rapamycin presented significantly more LC3B puncta (23 ± 1.6, *P* < 0.0001). In addition, the mRNA levels of the HR-associated genes *Brca1* and *Rad51* and the NHEJ-associated gene *Dnapk* were measured, and the results indicated that the *Rad51* and *Dnapk* mRNA levels were markedly increased in the oocytes of the rapamycin group, while the *Brca1* levels showed no significant differences between the two groups.

### Rapamycin regulated the indispensable Rad51-mediated HR to promote DSB repair during oocyte IVM

**Fig. 3:**
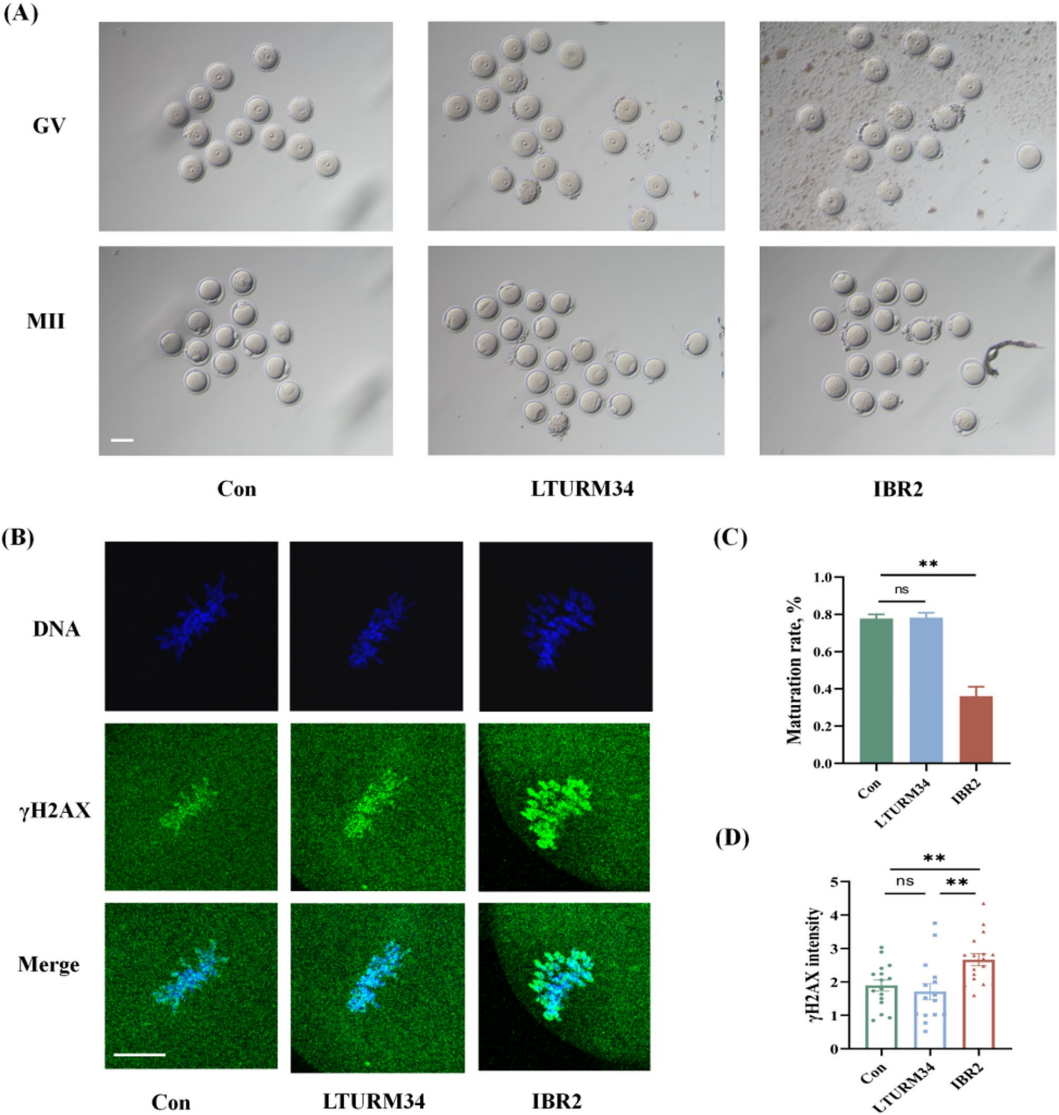
The impact of RAD51 inhibitor and DNAPK inhibitor on IVM oocytes. (**A**) The images of IVM oocytes treated with RAD51 inhibitor (IBR2) and DNAPK inhibitor (LTURM34). Scale bar: 50 μm. (**B**) The fluorescent images of γH2AX in IVM oocytes from the three groups. Scale bar: 10 μm. (**C**) The comparison of maturation rates of IVM oocytes treated with RAD51 inhibitor (IBR2) and DNAPK inhibitor (LTURM34), **P < 0.01. (**D**) The γH2AX fluorescence levels of IVM oocytes in control group and treated with IBR2 or LTURM34. **P < 0.01

To further confirm the roles of RAD51 and DNAPK in the DSB repair of IVM oocytes, IBR2 and LTURM34 were added into the medium to respectively inhibit RAD51 and DNAPK during oocyte IVM. The results, as shown in Fig. 3, indicated that the IBR2 significantly reduced maturation rate (36.1%±5.1% vs. 77.8%±2.2%, *P* = 0.0017) and increased DNA damage levels (2.7 ± 0.2 vs. 1.9 ± 0.2, *P* = 0.0087), while DNAPK inhibitor showed no effects on the development of IVM oocytes.

**Fig. 4:**
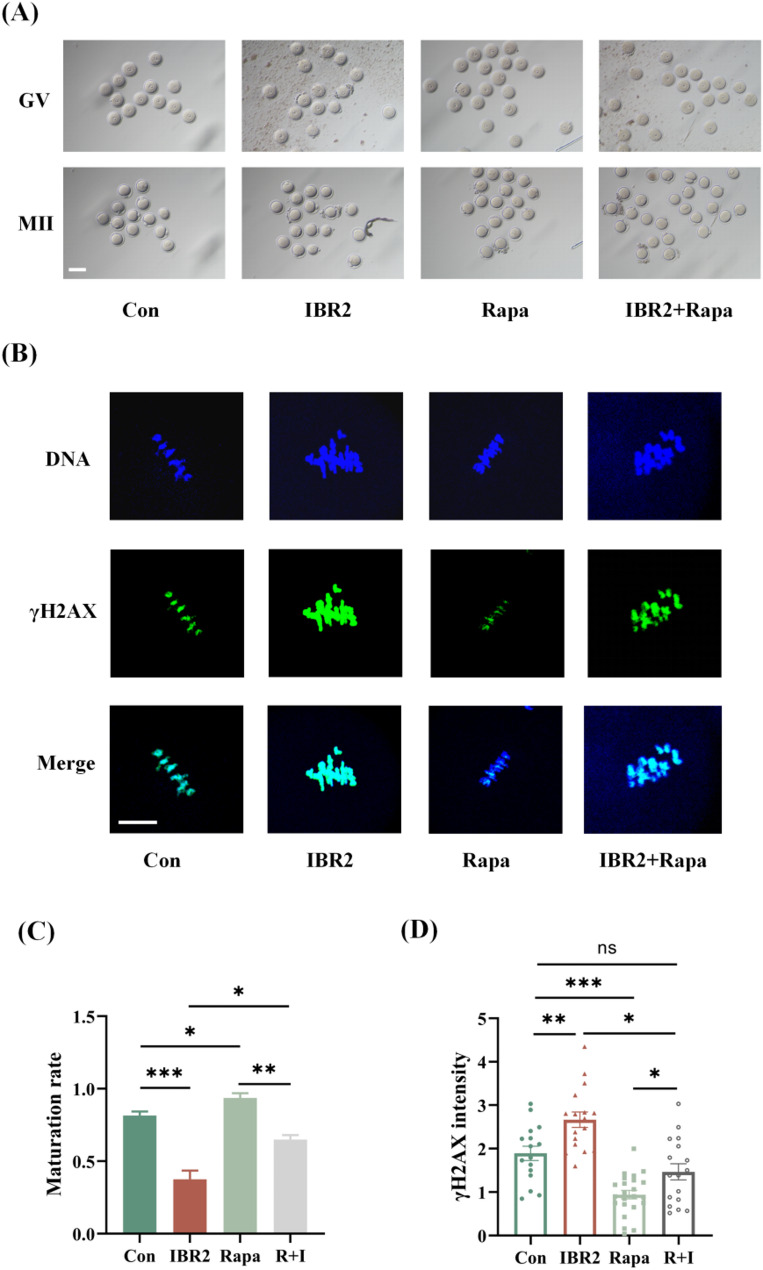
The impact of RAD51 inhibition on the improving effects of rapamycin in IVM oocytes. (**A**) Images of oocytes IVM from the control group, rapamycin group, IBR2 group, and R + I group. Scale bar: 50 μm. (**B**) γH2AX fluorescence staining of IVM oocytes from the four groups. (**C**) Comparison of the maturation rates of the four groups. (**D**) Comparison of the γH2AX fluorescence levels of the four groups. *P < 0.05; **P < 0.01; ***P < 0.001

Subsequently, the impact of IBR2 on the improving effect of rapamycin during oocyte IVM was evaluated by adding IBR2 and rapamycin into the medium, independently or simultaneously. As shown in Fig. 4, rapamycin significantly increased the maturation rate of IVM oocytes (93.7%±3.2% vs. 81.6%±2.7%, *P* = 0.0294). In the R + I group (adding rapamycin and IBR2 simultaneously), however, the maturation rate markedly decreased to 65.0%±3.1% (*P* = 0.0029). Additionally, oocytes from the rapamycin group showed reduced γH2AX fluorescence levels compared with those from the control group (0.9 ± 0.1 vs. 1.9 ± 0.2, *P* < 0.0001), while in the R + I group, the γH2AX fluorescence levels increased to 1.5 ± 0.2, with statistically significant difference (*P* = 0.0473).

## Discussion

In the current study, we investigated the protective effects of rapamycin on IVM oocytes, focusing on its ability to reduce DNA DSB and improve developmental competence. Our findings demonstrated that rapamycin significantly enhanced the maturation rates and developmental potential of IVM oocytes, as evidenced by increased 2PN rates and cleavage rates following ICSI. Importantly, we identified RAD51-mediated HR as the primary pathway through which rapamycin promotes DSB repair during oocyte IVM. These results provided novel insights into the mechanisms underlying rapamycin’s beneficial effects on oocyte quality and highlighted its potential as a therapeutic agent to optimize IVM outcomes.

Rapamycin is a macrolide metabolite produced by *Streptomyces hygroscopicus*, which was first identified in 1975, with antifungal and immunosuppressive effects [17, 18]. In accordance with other studies, our previous results have proved that rapamycin can affect the outcomes of oocyte IVM, which was largely determined by its concentration [7]. The increased 2PN and cleavage rates in the rapamycin-treated group suggested that rapamycin enhanced not only nuclear maturation but also cytoplasmic maturation, which is critical for subsequent embryonic development. However, the blastocyst formation rates did not differ significantly between the rapamycin and control groups, indicating that the beneficial effects conferred by rapamycin may be time-limited. Interestingly, our previous parthenogenetic activation experiments demonstrated that rapamycin could enhance blastocyst formation rates post-activation [7], implying that the presence of untreated paternal genetic material might compromise rapamycin’s protective effects on maternal DNA stability. In future studies, it would be valuable to further investigate the impact of rapamycin on sperm or embryonic development.

A key finding of this study was that rapamycin reduces DNA DSB levels in IVM oocytes without inducing autophagy at a concentration of 10 nM. This was particularly important because autophagy, while generally protective, could also lead to excessive degradation of cellular components and compromise oocyte quality if overactivated. Some previous literature has mainly focused on the autophagy activation effect of rapamycin in oocytes- the autophagy of oocytes in the rapamycin group was activated, leading to the reduced level of pro-apoptotic gene *Bax* and increased level of anti-apoptotic gene *Bcl-xL* [19]. Nevertheless, our data showed that 10 nM rapamycin did not increase LC3B puncta, a marker of autophagy, whereas a higher concentration (10 µM) significantly induces autophagy. This suggested that the beneficial effects of rapamycin on DSB repair were independent of its autophagy-activating properties, at least at the lower concentration used in this study.

More importantly, our study provided compelling evidence that RAD51-mediated HR was the primary pathway responsible for DSB repair in IVM oocytes. The significant upregulation of Rad51 mRNA levels in rapamycin-treated oocytes, coupled with the marked reduction in maturation rates and increased DSB levels upon RAD51 inhibition, underscored the critical role of HR in maintaining genomic integrity during oocyte maturation. In contrast, inhibition of DNA-PK, a key mediator of NHEJ, had no significant effect on oocyte maturation or DSB levels, suggesting that NHEJ played a minor role in DSB repair during IVM. These findings were consistent with previous studies showing that HR is the predominant DSB repair pathway in mammalian oocytes, whereas NHEJ is less active or potentially detrimental due to its error-prone nature [20, 21].

The observation that rapamycin’s beneficial effects were abolished by RAD51 inhibition further supported the hypothesis that rapamycin enhanced RAD51-mediated HR. This was consistent with some studies in cancer cells showing that rapamycin could modulate DNA repair pathways through the mTOR/AKT signaling axis [13, 14]. However, some other studies regarding cancer cells showed contradictory results [16]. This discrepancy may reflect cell type-specific differences in the regulation of DNA repair pathways. The autophagy level and DNA repair function in cancer cells were already abnormally elevated, and the research conclusions in different cancer cells were not completely consistent [22, 23], let al. one compared with normal somatic cells or IVM oocytes. In addition, the effect of rapamycin on DNA repair function may also be related to its concentration. In the above study on breast cancer cells [16], the author mentioned that the inhibitory effect of rapamycin on HR at a lower concentration (10 ng/ml) was weaker than that at 25 ng/ml, highlighting the need for further research to explore the optimal concentration and positive effects of rapamycin based on different cell types and clinical purposes.

Our results suggested that rapamycin could be used to improve IVM outcomes by enhancing oocyte quality and developmental potential, which have important implications for assisted reproductive technologies (ART). As for the clinical safety of rapamycin, the Food and Drug Administration (FDA) approved the use of rapamycin in the clinical prevention and treatment of renal transplant rejection in 1999. Later, other pharmacological effects of rapamycin and its derivatives were gradually discovered, and they were successively approved by the FDA for the treatment of advanced renal cancer, pancreatic progressive neuroendocrine tumor, lymphatic leiomyoma, breast cancer, tuberous sclerosis and other diseases alone or in combination with other drugs [24]. Moreover, the effectiveness and safety of rapamycin in anti-aging and prolonging lifespan have also been recognized [25]. Most recently, a cohort study demonstrated that 3 months of oral rapamycin treatment in infertile women with endometriosis was a beneficial strategy for improving in vitro fertilization-embryo transfer (IVF-ET) outcomes [26]. Taken together, rapamycin is a relatively safe drug in clinical practice, which provides a promising method to improve ART outcomes.

## Conclusion

In conclusion, our study demonstrates that rapamycin improves the developmental competence of IVM oocytes by reducing DNA DSB levels through the enhancement of RAD51-mediated HR. These findings not only advance our understanding of the molecular mechanisms regulating oocyte quality but also highlight the potential of rapamycin as a therapeutic agent to optimize IVM outcomes. Future research should focus on confirming the safety of rapamycin in offspring and exploring the broader applications of rapamycin in reproductive medicine.

## Electronic Supplementary Material

Below is the link to the electronic supplementary material.

Supplementary Material 1

## Data Availability

No datasets were generated or analysed during the current study.

## Abbreviations

IVM: In vitro maturation
PB1: First polar body
DSBs: Double-strand breaks
GVBD: Germinal vesicle breakdown
mTOR: Mammalian target of rapamycin
NHEJ: Non-homologous end joining
HR: Homologous recombination
ICSI: Intracytoplasmic sperm injection
ART: Assisted reproductive technologies
FDA: Food and Drug Administration
IVF-ET: In vitro fertilization-embryo transfer
