# Supplementary material for: Rapamycin reduces DNA damage of in vitro matured oocytes by promoting Rad51-mediated homologous recombination
Source: Reprod Biol Endocrinol. 2025 Jul 3;23:93. doi: 10.1186/s12958-025-01428-6 (PMC12224781; doi:10.1186/s12958-025-01428-6)
Supplement: Supplementary file 1 — Supplementary Material 1 [file 12958_2025_1428_MOESM1_ESM.docx]

**Supplemental Table 1 Primers used for real-time PCR**

| Gene | Primer sequence | Annealing temp. |
| --- | --- | --- |
| RAD51 | F: GGTCCACAGCCTATTTCA | 60℃ |
|  | R: TTATTAGTTCCTTCTTCGGT |  |
| BRCA1 | F: AGGCTTTGTCCATCATCT | 60℃ |
|  | R: GGTTTCCTATTGTCAGTTTT |  |
| DNAPK | F: GCCTTTTCGTCTAACCCG | 50℃ |
|  | R: ATCCTCCTTTTCTCAGCATT |  |
| GAPDH | F: ATTCAACGGCACAGTCAA | 60℃ |
|  | R: TTAGTGGGGTCTCGCTCC |  |
